# Fever, Leukocytosis, and Ulcerated Vulvar Lesions: An Atypical Presentation Concerning Behcet’s Disease

**DOI:** 10.7759/cureus.64159

**Published:** 2024-07-09

**Authors:** Austin Patrick Eisenberg, Brenden Pearce, Casey Anders, Victor Collier

**Affiliations:** 1 Internal Medicine, Grand Strand Medical Center, Myrtle Beach, USA

**Keywords:** herpetic lesions, general internal medicine, rheumatology, vulvar lesions, behcet’s syndrome

## Abstract

Behcet’s disease (BD) is a variable-vessel vasculitis commonly presenting in early adulthood with painful oral aphthous ulcers, genital ulcers, uveitis, pathergy, and skin lesions. The diagnosis of BD is made clinically based on criteria from the International Study Group (ISG) and the International Criteria for Behcet’s Disease (ICBD). Due to the wide constellation of symptoms BD can cause, it can be challenging to diagnose in an acute setting. Here, we discuss a patient who presented with a clinical picture of sepsis, with profound ulcerated vulvar and herpetiform oral mucosal lesions, that led us to a presumptive diagnosis of Behcet’s disease.

## Introduction

Behcet’s disease (BD), also known as Behcet’s syndrome, is an incompletely understood disease process involving autoimmune and autoinflammatory pathophysiology that can affect nearly any organ system [1]. It is one of two diseases classified as variable vessel vasculitis, the other being Cogan’s syndrome [2]. BD disease commonly presents in early adulthood with recurrent painful oral aphthous ulcers, genital ulcers, uveitis, pathergy, and skin lesions such as erythema nodosum. BD can also affect other organ systems, causing vascular thrombosis, gastrointestinal and neurologic manifestations, arthralgias, and arthritis [3]. Here, we discuss a female patient who presented with a clinical picture of sepsis, with profound ulcerated vulvar lesions and herpetiform oral mucosal lesions, which eventually led us to a presumptive diagnosis of Behcet’s disease.

## Case presentation

A 33-year-old female with a medical history significant for human papillomavirus (HPV), treated successfully in the past, presented to the emergency department (ED) due to worsening vaginal pain, swelling, and discharge. The symptoms began ten days prior. She tried an over-the-counter vaginal suppository without improvement. The patient noted increased swelling around her mandible, as well as painful cervical and axillary adenopathy. She reported fevers, chills, fatigue, and dysuria. Developing a sore throat and labial swelling led her to seek care at an urgent care clinic, where she received a diagnosis of a herpes simplex virus infection and a urinary tract infection. She was initially prescribed valacyclovir and cefdinir. Several days later, she was reevaluated at a different clinic; these medications were discontinued, and she was started on metronidazole. The patient is married and maintains a monogamous relationship with her husband.

**Figure 1:**
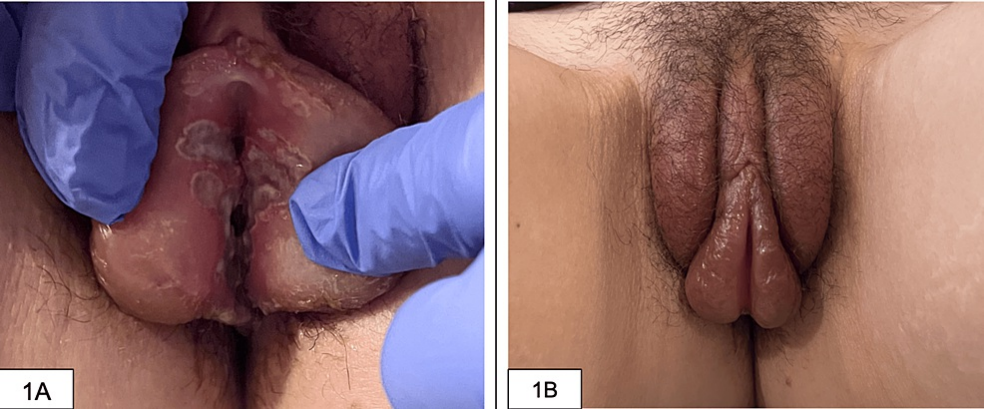
Physical examination A: ulcerated lesions along the patient’s vulva; B: significant swelling of the patient's labia.

Upon presentation to the ED, her vitals were notable for a temperature of 101.1 degrees Fahrenheit (38.4 degrees Celsius), a heart rate of 101 beats per minute, a respiratory rate of 18, a blood pressure of 98/52, and an oxygen saturation of 99% on room air. Initial lab work revealed a white blood cell (WBC) count of 17.1 K/mm3 (3.7-10.1 K/mm3) with a neutrophilic predominance, C-reactive protein of 19.4 mg/dL (0-0.99 mg/dL), erythrocyte sedimentation rate of 44 mm/hr (0-20 mm/hr), and a lactic acid level of 1 mmol/L (0.7-2.0 mmol/L). Testing for human immunodeficiency virus (HIV) antigen/antibody was negative; a urine pregnancy test was negative; and gonorrhea/chlamydia testing was negative. Abdominal and pelvic CT scans with contrast did not reveal any acute intra-abdominal abnormalities. Physical examination at this juncture demonstrated vesicles in the oral cavity and ulcerative lesions on the vulvar wall (Figures 1A, 1B). Due to severe pain, a speculum exam was deferred. As she met sepsis criteria on arrival, she was fluid resuscitated, and treatment was initiated with vancomycin, piperacillin-tazobactam, and acyclovir.

**Figure 2:**
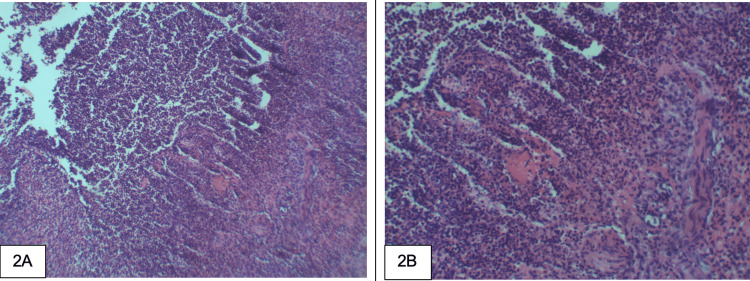
Biopsy results A: vulvar lesion in H&E stain at 10x magnification; B: 20x magnification, showing benign granulation tissue with marked acute inflammation.

Blood cultures and urine cultures remained negative, and her clinical picture remained unclear. Infectious etiologies became less likely without improvement on broad-spectrum antibiotics, and autoimmune disorders quickly moved up our differential. Antibiotics were discontinued, and intravenous (IV) methylprednisolone and colchicine were initiated. IV-methylprednisolone was changed to oral prednisone. A biopsy of the vulvar wall was obtained and revealed diffuse inflammation (Figures 2A, 2B). The patient responded well to the new medication regimen. At the time of discharge, her herpes simplex virus (HSV) culture result was pending, and she was discharged with valacyclovir, a prednisone taper, and colchicine. HSV culture resulted after discharge, showing no herpes simplex virus isolated. Arrangements were made for her to follow up with a rheumatologist for further evaluation.

## Discussion

In this report, we illustrate a likely initial presentation of Bechet’s disease (BD) in a 33-year-old female of Colombian descent. BD is a variable-vessel vasculitis that commonly presents in early adulthood with painful oral aphthous ulcers, genital ulcers, uveitis, pathergy, and skin lesions such as erythema nodosum [1]. Less commonly, it can cause arterial and venous thrombosis and thromboangiitis. Unlike other vasculitides, pulmonary and arterial aneurysms are unique to BD. BD can also involve the nervous and gastrointestinal systems [2]. 

The diagnosis of BD can be challenging in the outpatient setting, let alone in the acute inpatient setting. BD is a clinical diagnosis based on the Revised International Criteria, which includes recurrent oral and/or genital ulcerations, eye lesions, skin lesions, vascular lesions, and a positive pathergy test [2,3]. In our patient, this was likely the initial presentation of these symptoms. During our patient’s hospitalization, the diagnosis of BD could not be definitively made as the recurrence of symptoms is needed to meet diagnostic criteria. 

Behcet’s disease is frequently associated with individuals of Turkish, Japanese, and Middle Eastern descent [3,4]. However, Behcet's disease can affect any population, such as our patient, who was a female of Colombian descent. Amongst Latin American countries, the mean age at diagnosis of BD was 33 years, with 58% female patients [5]. Similar to our patient, the most common manifestations were oral and genital ulcers [5]. A study by Fernández-Ávila et al. described the prevalence of BD in Colombia as 1.1/100,000 inhabitants, with 68% being female [6]. 

The pathophysiology of Behcet's disease is not completely understood, although a strong genetic association with the HLA-B51 allele has been demonstrated repeatedly [4-7]. Inflammation is a driving force in the disease. Dysregulated cell-mediated immunity and increased Nuclear Factor 𝛋B (NF-𝛋B) signaling are thought to play a critical role in the pathogenesis of BD [8-10]. These pathways lead to increased macrophage activation, neutrophil chemotaxis, and phagocytosis, and thus, neutrophilic infiltrates are predominantly seen in biopsies of Bechet’s lesions [10], as demonstrated in Figures 2A, 2B. This increased activation of the pro-inflammatory pathways could explain our patient's initial systemic inflammatory response syndrome (SIRS)-positive presentation. SIRS is often associated with sepsis, though it’s important to keep a broad differential and be aware of atypical presentations of other disease processes.

## Conclusions

This case represents the significance of keeping autoimmune conditions on the differential when the clinical picture is muddied. Autoimmune disorders can present in a multitude of ways, mimicking other diagnoses for which we may already have an illness script. Our patient presented with an overt picture of sepsis secondary to a urogenital source, and despite appropriate treatment for that presumed diagnosis, she did not improve. This necessitated a diagnostic “time-out” and forced our team to evaluate her through the lens of other diagnoses. Although we were not able to definitively make the diagnosis of Behcet’s disease, we were able to provide relief for the presenting symptoms and establish her with a rheumatologist to ensure continuity of care.
